# Exploring Orthogonality between Halogen and Hydrogen Bonding Involving Benzene

**DOI:** 10.3390/molecules26237126

**Published:** 2021-11-25

**Authors:** Alessandra Forni, Rosario Russo, Giacomo Rapeti, Stefano Pieraccini, Maurizio Sironi

**Affiliations:** 1 Istituto di Scienze e Tecnologie Chimiche “Giulio Natta”—CNR, INSTM RU, Via Golgi 19, 20133 Milan, Italy; 2 Department of Chemistry, Università degli Studi di Milano, INSTM RU, Via Golgi 19, 20133 Milano, Italy; russorosario@itis-molinari.eu (R.R.); giacomo.rapeti@studenti.unimi.it (G.R.)

**Keywords:** halogen bonding, hydrogen bonding, orthogonal interactions, DFT calculations, halogen-π interaction

## Abstract

The concept of orthogonality between halogen and hydrogen bonding, brought out by Ho and coworkers some years ago, has become a widely accepted idea within the chemists’ community. While the original work was based on a common carbonyl oxygen as acceptor for both interactions, we explore here, by means of M06-2X, M11, *ω*B97X, and *ω*B97XD/aug-cc-PVTZ DFT calculations, the interdependence of halogen and hydrogen bonding with a shared π-electron system of benzene. The donor groups (specifically NCBr and H_2_O) were placed on either or the same side of the ring, according to a double T-shaped or a perpendicular geometry, respectively. The results demonstrate that the two interactions with benzene are not strictly independent on each other, therefore outlining that the orthogonality between halogen and hydrogen bonding, intended as energetical independence between the two interactions, should be carefully evaluated according to the specific acceptor group.

## 1. Introduction

Halogen bonding (XB), a noncovalent interaction where the halogen atom acts as electrophilic species [1], is nowadays a well-recognized molecular interaction with applications in biochemistry [2,3,4,5,6,7,8,9,10,11] and materials science [12,13], including non-linear optics [14,15,16] and liquid crystals [17,18,19]. This interaction, schematized as R–X···B (X = Cl, Br, or I; B = Lewis base; R = substituent), has been explained by the existence of a region of positive electrostatic potential, named σ-hole, on the outermost surface of the covalently-bonded halogen atom and narrowly confined on the elongation of the R–X covalent bond axis [20]. Its presence has been recently demonstrated through valence bond spin-coupled calculations [21], allowing to get a rigorous ab initio validation of the qualitative models previously proposed [20]. The key role of the σ-hole in activating XB has been particularly emphasized by molecular mechanics/molecular dynamics simulations of halogen-bonded ligand–protein systems. In fact, such calculations were able to reproduce the experimentally observed structural features only if the charge anisotropy around the halogen atom was correctly described through introduction of a positively charged particle mimicking the σ-hole [22,23,24,25,26,27]. The presence of the σ-hole has also been demonstrated by experimental charge density studies [28,29,30,31,32,33,34,35].

Analysis of crystal structures of halogenated molecules has revealed that XB often acts in a cooperative way with hydrogen bonding (HX) [36,37,38,39]. In particular, Ho and coworkers reported that halogen and hydrogen bonds can be geometrically perpendicular to and energetically independent on each other, when the involved X and H donor atoms interact with the same carbonyl group in protein–ligand complexes [40]. The authors then proposed the concept of XB/HB orthogonality, paving the way for the development of new strategies aimed at the rational design of halogenated ligands as drugs [36]. 

Prompted by the conclusions obtained by Ho and coworkers on the CO···X/H orthogonality [40], we have considered the possibility to extend this concept to the case where the halogen and hydrogen donor atoms share a common benzene π-electron system as bonding acceptor. In previous studies [41,42,43], we extensively investigated from a theoretical point of view the XB established between a series of halogenated molecules (NCX or PhX where X = F, Cl, Br, I) and the aromatic system of benzene in the T-shaped configuration, an interaction rather ubiquitous in biological systems [44,45]. Here, the more appropriated computational protocols proposed in our previous studies are used to investigate the simultaneous interaction of NCBr and H_2_O, two relatively strong XB and HB donors, respectively, with a common π-electron system of benzene. To this purpose, two geometrical approaches have been examined, that is, a ‘double T-shaped’ one, where NCBr and H_2_O, both in T-shaped configuration with respect to benzene, point to the ring from opposite sides; and a ‘perpendicular’ approach, where NCBr and H_2_O lie on the same side of the ring forming a right angle with the center of the ring. Of course, the concept of orthogonality we want here to explore refers uniquely to the energetical independence of the two interactions rather than to the geometrical arrangement of the interacting species, which strictly applies only to the ‘perpendicular’ approach. To provide solid support to our conclusions, different functionals have been used for this analysis, that is M06-2X, M11, and *ω*B97X, chosen among the better performing on the basis of our previous investigation on the NCX···π XB [42]. Owing to the demonstrated importance of the dispersion forces, besides the electrostatic ones, in describing the RX···π interaction [42], additional calculations were performed with a DFT-D functional, *ω*B97XD, which explicitly includes *a posteriori* dispersion correction.

## 2. Results

### 2.1. Double T-Shaped Configuration

The simultaneous formation of XB and HB with benzene, where the respective donors are located on opposite sides with respect to the plane of the ring, represents a quite common motif in organic crystal structures, as denoted by a survey of the Cambridge Structural Database (CSD version 5.42, February 2021) [46]. Up to 962 hits were in fact obtained by searching for organic compounds containing A–X···c(Ph)···H–B fragments (being A and B generic atoms, c(Ph) the geometrical centroid of a phenyl ring, and X = Cl, Br or I) with X···c(Ph) and c(Ph)···H distances in the 3.0–4.0 Å and 2.0–3.0 Å range, respectively, and both A–X···c(Ph) and X···c(Ph)···H angles in the 150°–180° range. 

**Figure 1:**
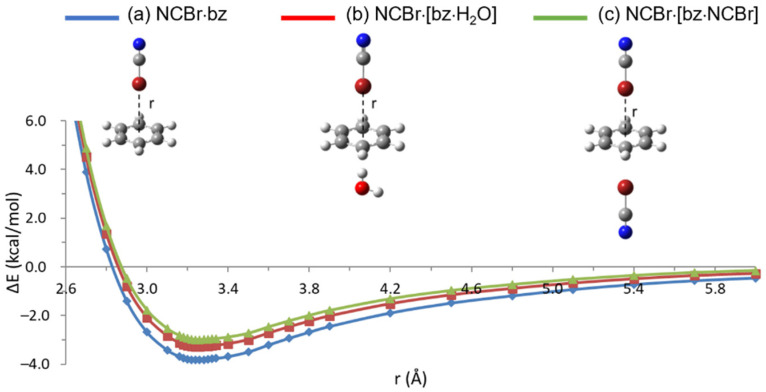
Binding energy curves, ΔE, vs. Br distance from the center of benzene ring, r, computed for (**a**) NCBr·bz (blue line), (**b**) NCBr·[bz·H_2_O] (red line) and (**c**) NCBr·[bz·NCBr] (green line) systems in the T-shaped approach at the M06-2X/aug-cc-PVTZ level of theory.

**Figure 2:**
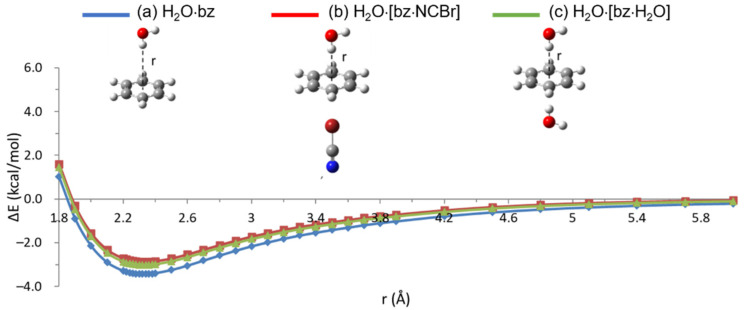
Binding energy curves, ΔE, vs. (HO)H distance from the center of benzene ring, r, computed for (**a**) H_2_O·bz (blue line), (**b**) H_2_O·[bz·NCBr] (red line) and (**c**) H_2_O·[bz·H_2_O] (green line) systems in the T-shaped approach at the M06-2X/aug-cc-PVTZ level of theory.

A first set of calculations were performed on dimers of NCBr or H_2_O with benzene (or bz) in T-shaped configuration (see structure (a) in Figure 1 and Figure 2, respectively), with the Br or the H atom, respectively, approaching the center of the ring. The binding energy curves, computed at the M06-2X/aug-cc-PVTZ level of theory, are plotted in Figure 1 and Figure 2 (see Figures S1–S6 for the M11, *ω*B97X, and *ω*B97XD binding energy curves and Tables S1–S8 for the ΔE vs. r numerical values). Comparing halogen and hydrogen bonds, the four examined functionals display the same trend in the values of equilibrium distances, r_eq_, and interaction energies, ΔE_XB_ and ΔE_HB_ for XB and HB, respectively (see Table 1). For both the NCBr·bz and H_2_O·bz optimized dimers, in fact, M06-2X provides the higher ΔE_XB_ and ΔE_HB_ interaction energies, respectively, which correspond to the shorter intermolecular distances. The M11 functional gives the lower interaction energies, with differences in ΔE_XB_ and ΔE_HB_ amounting to 0.35 and 0.42 kcal/mol (i.e., 9.1 and 12.2%), respectively, compared to the M06-2X values. Finally, the *ω*B97X and *ω*B97XD functionals provide the longer equilibrium distances and energy values intermediate between the M06-2X and the M11 ones. Comparing the two related *ω*B97X and *ω*B97XD hybrid functionals, it is found that the empirical dispersion correction does not provide any significant variation in both equilibrium distances and energies. Interestingly, however, while equilibrium distances of both interactions are slightly elongated when explicitly including dispersion, the corresponding energies undergo different, though very small, variations according to the HB or XB interaction. In the first case, in fact, ΔE increases in magnitude, as generally obtained going from DFT to DFT-D calculations on non-covalently bound systems [47,48], while for the XB interaction the opposite trend is observed, suggesting that dispersion effects are somehow taken into account in the *ω*B97X functional, too. 

Finally, it should be noted that the considered T-shaped approach represents a constrained path enabling to uniquely fix the dimers’ geometry for subsequent calculations on trimers. As previously reported for RX···π XB systems [41], unconstrained geometry optimization of such dimers leads to a breakdown of the T-shaped geometry of the monomers, the RX molecule being slightly out from the perpendicularity with respect to the benzene plane and pointing towards a CC benzene bond rather than to the center of the ring. However, geometry optimization of the NCBr·bz and H_2_O·bz dimers here examined, performed at the *ω*B97XD/aug-cc-PVTZ level of theory, results in absolute minima, confirmed by frequency calculations, which are only 0.20 and 0.50 kcal/mol (for XB and HB, respectively) more stable than the constrained T-shaped configuration (see the minimum energy geometries in Figure S7). This denotes a rather flat potential energy surface for the NCBr·bz and H_2_O·bz interacting systems, indicating that the chosen T-shaped configuration does not represent in any way a too strong assumption affecting the subsequent results.

Keeping the optimized dimers fixed in their respective T-shaped energy minima, we then computed the binding energy curves corresponding to the T-shaped approach of either NCBr to bz·H_2_O or H_2_O to bz·NCBr from the opposite site with respect to NCBr or H_2_O, respectively, according to a ‘double T-shaped’ geometry (see structure (b) in Figure 1 and Figure 2, respectively). The binding energies of the formed NCBr·[bz·H_2_O] and H_2_O·[bz·NCBr] trimers have been computed as the difference between the total energy and the sum of the contributions deriving from the incoming molecule and that of the fixed hydrogen- or halogen-bonded complex. Of course, such two-step formation of the trimer does not reproduce the real situation, where XB and HB are expected to be concomitantly established during the aggregation process, but it allows to separately evaluate the contributions of the two interactions.

Comparison between the ΔE_XB_ values obtained for the NCBr·bz dimer and those determined for the NCBr·[bz·H_2_O] trimer should provide an indication about the orthogonality between the two interactions: if no significant ΔE_XB_ variations are observed, XB and HB can be considered orthogonal. As a countercheck, the results obtained for H_2_O·bz and H_2_O·[bz·NCBr] should lead to comparable conclusions. 

The results are collected in Table 1 and the binding energy curves obtained by M06-2X calculations are plotted in Figure 1 and Figure 2 (see Figures S1–S6 for the M11, *ω*B97X, and *ω*B97XD binding energy curves and Tables S1–S8 for the ΔE vs. r numerical values). It is found that the minimum energy geometry of XB is only slightly perturbed when NCBr interacts with [bz·H_2_O] rather than with bz alone, with elongations lying within the step size used to build up the curves (0.02 Å in the minimum energy region). Even lower elongations are obtained for HB. Considering the interaction energies, however, non-negligible variations are obtained comparing the same interaction in the dimer and in the trimer. For example, looking at the results obtained with the M11 functional, providing the larger variations, ΔE_XB_ decreases in absolute value from −3.48 to −2.90 kcal/mol and ΔE_HB_ from −3.01 to −2.43, with a comparable percentage reduction of 16.7 (ΔE_XB_) and 19.3% (ΔE_HB_). These results are confirmed by M06-2X, *ω*B97X, and *ω*B97XD calculations, providing percentage reductions of 14.4 (ΔE_XB_), 16.0% (ΔE_HB_); 13.6 (ΔE_XB_), 15.9% (ΔE_HB_); and 12.8 (ΔE_XB_), 14.4% (ΔE_HB_), respectively. The destabilization associated with the trimer formation indicates a strong perturbation of the whole π-electron system of benzene within the dimer, allowing to conclude that XB and HB on either side of benzene are not strictly orthogonal interactions.

The slightly greater destabilization of HB with respect to XB in the presence of the other perturbing interaction on the other side of benzene should be imputed to the here considered NCBr·bz XB, which is slightly stronger (by 0.40, 0.47, 0.53, or 0.41 kcal/mol according to the M06-2X, M11, *ω*B97X, or *ω*B97XD functional) than the H_2_O·bz HB. This observation is supported by additional calculations on ‘purely’ halogen-bonded or hydrogen-bonded trimers, where the same molecule approaches the benzene ring from either side, resulting in double T-shaped NCBr·[bz·NCBr] or H_2_O·[bz·H_2_O] systems (see structure (c) in Figure 1 and Figure 2, respectively). A quite different behavior is observed for the halogen-bonded and the hydrogen-bonded trimers (see Table 1, Figure 1, Figure 2 and Figures S1–S6 for the M06-2X, M11, *ω*B97X, and *ω*B97XD binding energy curves, respectively, and Tables S1–S8 for the ΔE vs. r numerical values). In fact, looking again at the M11 results, calculations provide a much stronger reduction of the XB interaction energy with respect to that computed for the HB one, with percentage reductions equal to 24.7 (ΔE_XB_) and 14.6% (ΔE_HB_). Similarly, M06-2X, *ω*B97X, and *ω*B97XD calculations provide percentage reductions of 21.9 (ΔE_XB_), 12.0% (ΔE_HB_); 20.4 (ΔE_XB_), 12.1% (ΔE_HB_); 18.9 (ΔE_XB_), 11.0% (ΔE_HB_), respectively. Such results can be explained by considering that the π-electron system of benzene is perturbed in a greater extent by the stronger NCBr·bz XB with respect to the weaker H_2_O·bz HB.

### 2.2. Perpendicular Configuration

**Figure 3:**
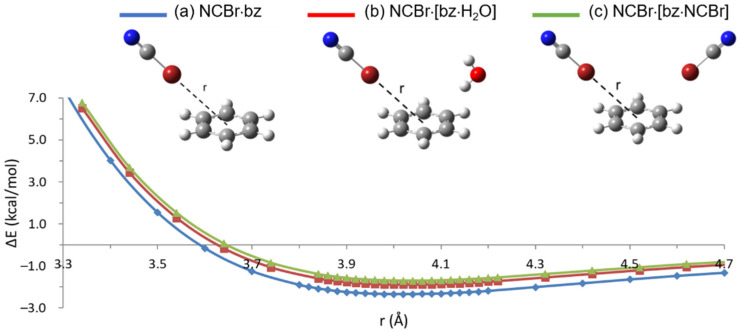
Binding energy curves, ΔE, vs. Br distance from the center of benzene ring, r, computed for (**a**) NCBr·bz (blue line), (**b**) NCBr·[bz·H_2_O] (red line), and (**c**) NCBr·[bz·NCBr] (green line) systems in the perpendicular approach at the M06-2X/aug-cc-PVTZ level of theory.

**Figure 4:**
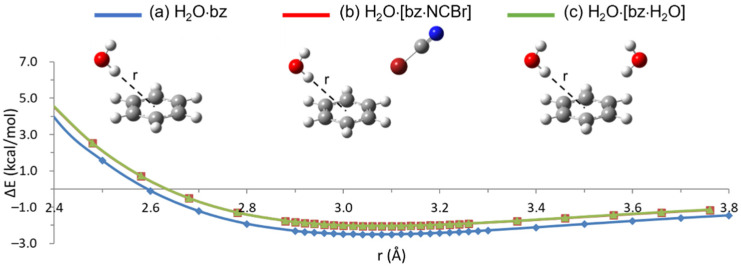
Binding energy curves, ΔE, vs. (HO)H distance from the center of benzene ring, r, computed for (**a**) H_2_O·bz (blue line), (**b**) H_2_O·[bz·NCBr] (red line), and (**c**) H_2_O·[bz·H_2_O] (green line) systems in the perpendicular approach at the M06-2X/aug-cc-PVTZ level of theory.

To explore the configuration where both NCBr and H_2_O molecules lie on the same side of the benzene ring, perpendicular to each other, preliminary M06-2X/aug-cc-PVTZ calculations have been performed on the halogen- and hydrogen-bonded dimers with benzene, aimed at establishing the constrained path of minimum energy towards the center of the ring. Binding energy curves were calculated by moving either NCBr or the interacting OH group along the line through the center of the ring and forming a 45° angle with the benzene plane. Both approaches where the projection of the line on this plane crosses one carbon atom or the center of a CC bond (see Figures S8 and S9, respectively) have been examined. Moreover, in the case of the hydrogen-bonded dimer, the non-interacting hydrogen atom was directed either inward or outward with respect to the benzene ring. As shown in Table S9, the more stable approach was found to be along the center of a CC bond for both dimers, with the non-interacting H atom of water outwards with respect to the benzene ring (see structure (a) in Figure 3 and Figure 4 for the NCBr·bz and H_2_O·bz dimers, respectively). Such an approach was then chosen for all the subsequent calculations. 

As expected, the resulting ΔE values (see Table 2, Figure 3, Figure 4 and Figures S10–S15 for the M06-2X, M11, *ω*B97X, and *ω*B97XD binding energy curves, respectively, and Tables S10–S17 for the ΔE vs. r numerical values) are lower than those computed for the T-shaped configuration, because such a ‘bent’ approach is even farther, compared to the T-shaped one, from the minimum energy path (see Figure S7). However, it is interesting to note that XB is now slightly less stable (by 0.17, 0.20, 0.13, and 0.25 kcal/mol according to the M06-2X, M11, *ω*B97X, and *ω*B97XD functional, respectively) than HB, differently from what derived for the T-shaped configuration. Such a result could be explained by the greater directionality of XB with respect to HB, due the more restricted positive region of the electrostatic potential on the halogen atom, compared to that hemispherically distributed around the hydrogen atom [49]. In the present approach, the NCBr molecule is then more affected, with respect to H_2_O, by being quite far from the minimum energy path.

Compared with the results obtained on dimers in T-shaped configuration, the higher interaction energies are obtained with the *ω*B97X functional, while M11 still provides the lower ΔE values (see Table 2). Moreover, the examined functionals provide much more different interaction energies despite rather similar equilibrium distances. The variations from the *ω*B97X values to the M11 ones amount to 0.68 (ΔE_XB_) and 0.61 (ΔE_HB_) kcal/mol, corresponding to 27.5 and 23.5% reductions with respect to the higher *ω*B97X values. Such discrepancies are much higher than those (9.1 and 12.2%) determined for the T-shape approach. Considering that, in the two different approaches, we are dealing with the same molecules in different orientations, it may be hypothesized that the larger discrepancies observed for the bent dimers could be ascribed to the fact that this orientation is more outside, with respect to the T-shaped configuration, the minimum energy one, implying a stronger angular dependent energy bias [50].

We then computed the binding energy curves corresponding to the perpendicular approach, on the same side of the benzene ring, of either NCBr to bz·H_2_O or H_2_O to bz·NCBr, keeping the dimers fixed in their respective energy minima (see structure (b) in Figure 3 and Figure 4 for the NCBr·bz and H_2_O·bz dimers, respectively). As obtained for the double T-shaped configuration, the minimum energy geometry for both interactions is only slightly perturbed when NCBr or H_2_O interact with the dimer rather than with benzene alone (see Table 2). However, the corresponding interaction energies undergo even larger variations compared with those obtained for the double T-shaped approach: according to M11, M06-2X, *ω*B97X, and *ω*B97XD calculations, ΔE_XB_ and ΔE_HB_ values decrease by 27.3, 19.7, 19.0, 17.4%, and 24.1, 18.7, 17.7, 16.1%, respectively (to be compared with the corresponding values, 16.7, 14.4, 13.6, 12.8% and 19.3, 16.0, 15.9, 14.4%, of the double T-shaped configuration). Also in this case, the destabilization associated with the trimer formation indicates that XB and HB, perpendicularly approaching benzene from the same side, are not orthogonal interactions. The larger ΔE variations obtained for the perpendicular configuration denote an even greater interdependence between the two interactions with respect to that derived for the double T-shaped one.

The larger destabilization obtained for XB with respect to HB reflects the above-mentioned weaker NCBr·bz XB with respect to the H_2_O·bz HB in such a bent approach. Interestingly, however, calculations on NCBr·[bz·NCBr] halogen-bonded or H_2_O·[bz·H_2_O] hydrogen-bonded trimers in perpendicular configuration (see structure (c) in Figure 3 and Figure 4, respectively) provide again larger destabilization for XB with respect to HB, similar to what determined for the double T-shaped configuration. ΔE_XB_ and ΔE_HB_ decrease in fact by 37.4, 27.4, 25.9, 23.0%, and 24.6, 19.5, 18.1, 16.9% according to M11, M06-2X, *ω*B97X, and *ω*B97XD calculations, respectively, compared with the values of the dimers. This suggests a strong interdependence between the two perpendicular halogen-bonded interactions with benzene, despite their weakness with respect to the analogues hydrogen-bonded ones.

## 3. Materials and Methods

DFT geometry optimizations were performed by constraining the interacting molecules to the prefixed (T-shaped and double T-shaped; bent by 45° with respect to the benzene plane and perpendicular) geometries on the basis set superposition error (BSSE)-free potential energy surface. The distance of the Br or H atom from the center of the ring was varied from either 1.8 to 6.0 Å (T-shaped approaches) or 3.3 to 4.7 Å (perpendicular approaches with NCBr) or 2.4 to 3.8 Å (perpendicular approaches with H_2_O) with 0.1 Å step except for the region of minimum energy where the step size was reduced to 0.02 Å. Calculations were performed with the range-separated or long-range corrected GGA functional *ω*B97X [51], the hybrid meta-GGA functional M06-2X [52], and the range-separated hybrid meta-GGA functional M11 [53]. They were chosen based on their optimal performance to reproduce both interaction energies and geometry of benchmark CCSD(T)/CBS values, according to our previous studies [41,42]. Additional calculations were performed with *ω*B97XD [54] to check the effect of explicitly including dispersion corrections into the *ω*B97X functional. For these latter calculations, slightly reduced ranges of distances, including the minima, have been explored. In all cases, a large pruned integration grid (99 radial shells and 590 angular points per shell) was used to avoid artifacts associated with numerical integration procedures, as evidenced by previous investigation on the sensitivity of functionals to the size of the integration grid [55]. All calculations were performed with the aug-cc-PVTZ basis set, using the Gaussian 16 Revision A suite of programs [56].

## 4. Conclusions

The interdependence between halogen bonding and hydrogen bonding with a common π-electron system of benzene has been investigated by DFT calculations, using as donor molecules NCBr and H_2_O, respectively. Four different functionals, selected among the best performing based on previous investigation on the RX···π XB, namely M06-2X, M11, *ω*B97X, and *ω*B97XD, have been used to build up binding energy curves for both dimeric (i.e., NCBr·bz and H_2_O·bz) and trimeric (i.e., NCBr·[bz·H_2_O] and H_2_O·[bz·NCBr]) units. Two different configurations of the donor molecules with respect to benzene have been explored, i.e., a double T-shaped and a perpendicular one, where NCBr and H_2_O point towards the center of the ring from either opposite sides or the same side, perpendicularly to each other, respectively. In both cases, comparison between the interaction energies at the equilibrium distances computed for the trimers and the corresponding dimers indicates, for all the adopted functionals, that the two interactions cannot be considered strictly orthogonal, in particular as far as the perpendicular approach is concerned.

## Figures and Tables

**Table 1:** Equilibrium distances r_eq_ (Å) and interaction energies ΔE_XB_ and ΔE_HB_ (kcal/mol) for dimers and trimers in T-shaped and double T-shaped configurations, respectively.

Functional	NCBr·bz		NCBr·[bz·H_2_O]		NCBr·[bz·NCBr]	
	r_eq_	ΔE_XB_	r_eq_	ΔE_XB_	r_eq_	ΔE_XB_
M06-2X	3.24	−3.83	3.26	−3.28 (0.55) ^a^	3.26	−2.99 (0.84) ^a^
M11	3.32	−3.48	3.34	−2.90 (0.58) ^a^	3.34	−2.62 (0.86) ^a^
*ω*B97X	3.40	−3.67	3.42	−3.17 (0.50) ^a^	3.42	−2.92 (0.75) ^a^
*ω*B97XD	3.46	−3.60	3.48	−3.14 (0.46) ^a^	3.48	−2.92 (0.68) ^a^
	**H_2_O**·**bz**		**H_2_O**·**[bz**·**NCBr]**		**H_2_O**·**[bz**·**H_2_O]**	
	**r_eq_**	Δ**E_HB_**	**r_eq_**	Δ**E_HB_**	**r_eq_**	Δ**E_HB_**
M06-2X	2.32	−3.43	2.34	−2.88 (0.55) ^b^	2.34	−3.02 (0.41) ^b^
M11	2.34	−3.01	2.34	−2.43 (0.58) ^b^	2.34	−2.57 (0.44) ^b^
*ω*B97X	2.42	−3.14	2.42	−2.64 (0.50) ^b^	2.42	−2.76 (0.38) ^b^
*ω*B97XD	2.46	−3.19	2.46	−2.73 (0.46) ^b^	2.46	−2.84 (0.35) ^b^

^a^ In parentheses, the increase with respect to the NCBr·bz ΔE_XB_ value. ^b^ In parentheses, the increase with respect to the H_2_O·bz ΔE_HB_ value.

**Table 2:** Equilibrium distances r_eq_ (Å) and interaction energies ΔE_XB_ and ΔE_HB_ (kcal/mol) for dimers and trimers in perpendicular configuration.

Functional	NCBr·bz		NCBr·[bz·H_2_O]		NCBr·[bz·NCBr]	
	r_eq_	ΔE_XB_	r_eq_	ΔE_XB_	r_eq_	ΔE_XB_
M06-2X	4.02	−2.34	4.02	−1.88 (0.46) ^a^	4.04	−1.70 (0.64) ^a^
M11	4.06	−1.79	4.08	−1.30 (0.49) ^a^	4.10	−1.12 (0.67) ^a^
*ω*B97X	4.09	−2.47	4.11	−2.00 (0.47) ^a^	4.11	−1.83 (0.64) ^a^
*ω*B97XD	4.17	−2.30	4.17	−1.90 (0.40) ^a^	4.19	−1.77 (0.53) ^a^
	**H_2_O**·**bz**		**H_2_O**·**[bz**·**NCBr]**		**H_2_O**·**[bz**·**H_2_O]**	
	**r_eq_**	Δ**E_HB_**	**r_eq_**	Δ**E_HB_**	**r_eq_**	Δ**E_HB_**
M06-2X	3.06	−2.51	3.08	−2.04 (0.47) ^b^	3.08	−2.02 (0.49) ^b^
M11	3.08	−1.99	3.10	−1.51 (0.48) ^b^	3.10	−1.50 (0.49) ^b^
*ω*B97X	3.08	−2.60	3.10	−2.14 (0.46) ^b^	3.10	−2.13 (0.47) ^b^
*ω*B97XD	3.12	−2.55	3.14	−2.14 (0.41) ^b^	3.14	−2.11 (0.43) ^b^

^a^ In parentheses, the increase with respect to the NCBr·bz ΔE_XB_ value. ^b^ In parentheses, the increase with respect to the H_2_O·bz ΔE_HB_ value.

## Data Availability

The data presented in this study are available in supplementary materials.

## Supplementary Materials

The following are available online, Figures S1–S6 and S10–S15: Plots of binding energy curves, Figure S7: Fully optimized geometries of the NCBr·bz and H_2_O·bz dimers, Figures S8 and S9: Bent approaches of NCBr and H_2_O towards the center of benzene ring, Tables S1–S8 and S10–S17: Binding energies values, Table S9: Equilibrium distances and interaction energies for NCBr·bz and H_2_O·bz dimers in the 45° approaches.

## Sample Availability

Samples of the compounds are not available from the authors.
