# Supplementary material for: Exploring Orthogonality between Halogen and Hydrogen Bonding Involving Benzene
Source: Molecules. 2021 Nov 25;26(23):7126. doi: 10.3390/molecules26237126 (PMC8659280; doi:10.3390/molecules26237126)
Supplement: Supplementary file 1 [file molecules-26-07126-s001.zip › molecules-1447008-supplementary.pdf]

molecules

MDPI

Exploring Orthogonality between Halogen and Hydrogen Bonding Involving Benzene

Alessandra Forni [1,*], Rosario Russo [2], Giacomo Rapeti [2], Stefano Pieraccini [1,2,*] and Maurizio Sironi [1,2,*]

[1] Istituto di Scienze e Tecnologie Chimiche "Giulio Natta" – CNR and INSTM RU, via Golgi 19, 20133 Milan, Italy
[2] Department of Chemistry, Università degli Studi di Milano and INSTM RU, via Golgi 19, 20133 Milano, Italy
* Correspondence: alessandra.forni@scitec.cnr.it (A.F.); stefano.pieraccini@unimi.it (S.P.); maurizio.sironi@unimi.it (M.S.)

Supplementary Material

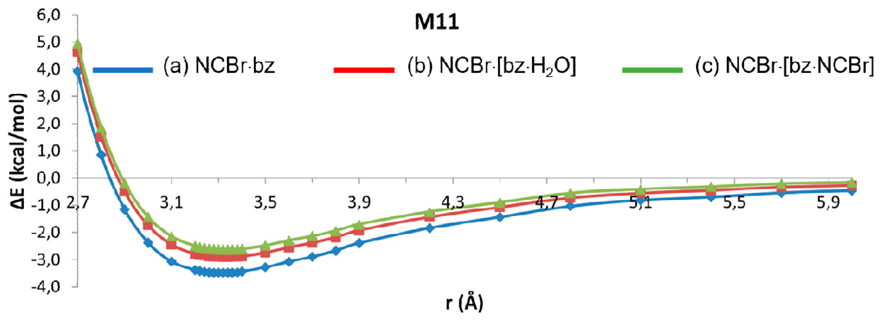

Figure S1. Binding energy curves, ΔE, vs. Br distance from the center of benzene ring, r, computed for (a) NCBr·bz (blue line), (b) NCBr·[bz·H_2O] (red line) and (c) NCBr·[bz·NCBr] (green line) systems in the T-shaped approach at the M11/aug-cc-PVTZ level of theory.

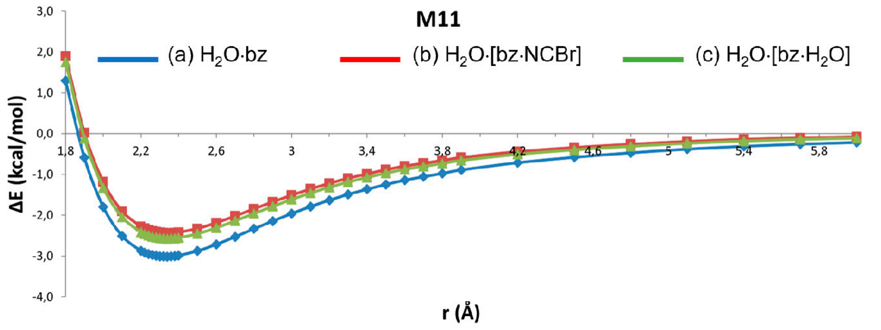

Figure S2. Binding energy curves, ΔE, vs. (HO)H distance from the center of benzene ring, r, computed for (a) H_2O·bz (blue line), (b) H_2O·[bz·NCBr] (red line) and (c) H_2O·[bz·H_2O] (green line) systems in the T-shaped approach at the M11/aug-cc-PVTZ level of theory.

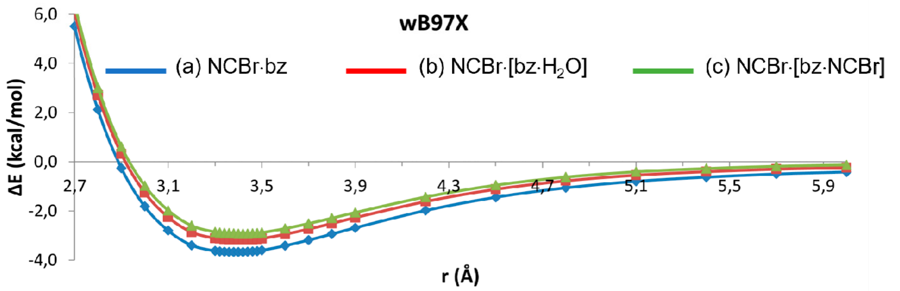

Figure S3. Binding energy curves, ΔE, vs. Br distance from the center of benzene ring, r, computed for (a) NCBr·bz (blue line), (b) NCBr·[bz·H_2O] (red line) and (c) NCBr·[bz·NCBr] (green line) systems in the T-shaped approach at the *ω*B97X/aug-cc-PVTZ level of theory.

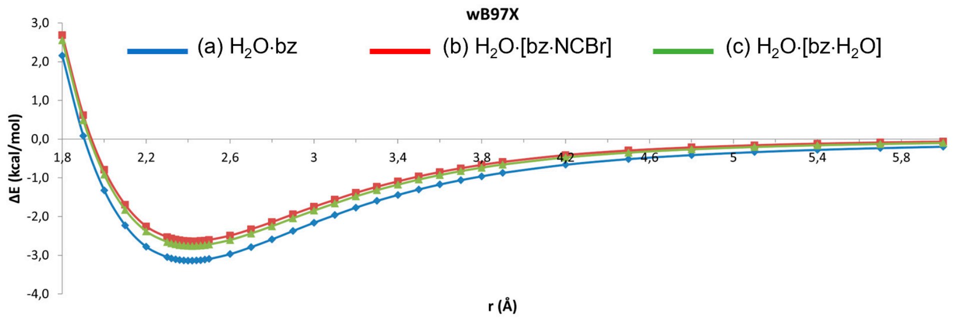

Figure S4. Binding energy curves, ΔE, vs. (HO)H distance from the center of benzene ring, r, computed for (a) H_2O·bz (blue line), (b) H_2O·[bz·NCBr] (red line) and (c) H_2O·[bz·H_2O] (green line) systems in the T-shaped approach at the *ω*B97X/aug-cc-PVTZ level of theory.

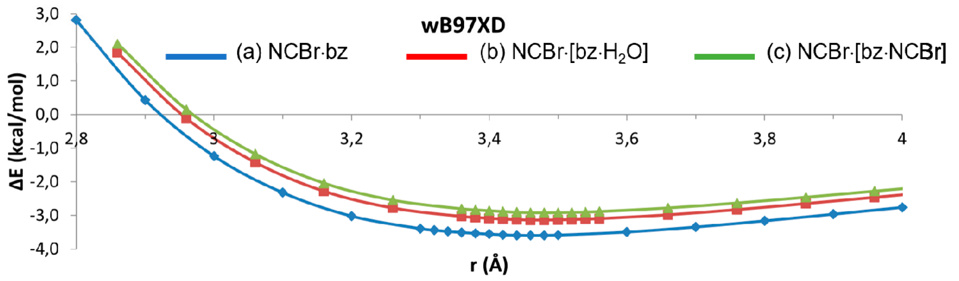

Figure S5. Binding energy curves, ΔE, vs. Br distance from the center of benzene ring, r, computed for (a) NCBr·bz (blue line), (b) NCBr·[bz·H_2O] (red line) and (c) NCBr·[bz·NCBr] (green line) systems in the T-shaped approach at the *ω*B97XD/aug-cc-PVTZ level of theory.

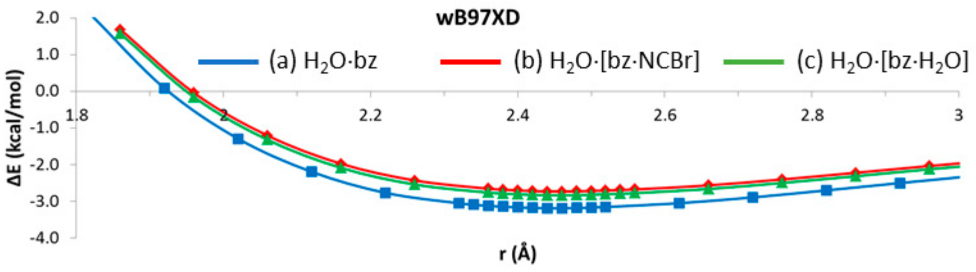

Figure S6. Binding energy curves, ΔE, vs. (HO)H distance from the center of benzene ring, r, computed for (a) H_2O·bz (blue line), (b) H_2O·[bz·NCBr] (red line) and (c) H_2O·[bz·H_2O] (green line) systems in the T-shaped approach at the *ω*B97XD/aug-cc-PVTZ level of theory.

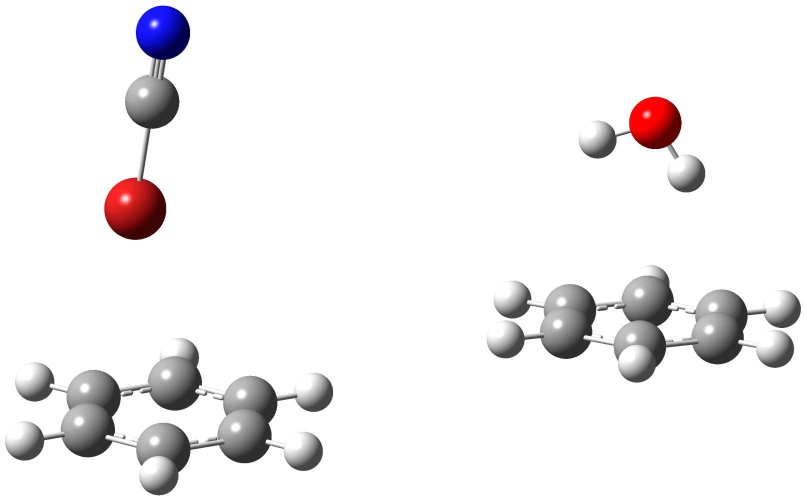

Figure S7. *ω*B97XD/aug-cc-PVTZ fully optimized geometries of the NCBr·bz (left) and H_2O·bz (right) dimers.

Table S1. Binding energy, ΔE, vs. Br distance from the center of benzene ring, r, computed for (a) NCBr·bz, (b) NCBr·[bz·H_2O] and (c) NCBr·[bz·NCBr] systems in the T-shaped approach at the M06-2X/aug-cc-PVTZ level of theory.

r (Å)	ΔE (kcal/mol)		
	(a) NCBr·bz	(b) NCBr·[bz·H_2O]	(c) NCBr·[bz·NCBr]
1.8	171.7322	172.5443	173.0282
1.9	129.8621	130.6326	131.1122
2	96.7615	97.5063	97.9688
2.1	70.8899	71.6055	72.0583
2.2	50.7751	51.4770	51.9042
2.3	35.3370	36.0170	36.4322
2.4	23.6149	24.2869	24.6784
2.5	14.8787	15.5343	15.9134
2.6	8.4525	9.1019	9.4616
2.7	3.8912	4.5249	4.8744
2.8	0.7301	1.3577	1.6900
2.9	-1.4017	-0.7917	-0.4685
3	-2.6810	-2.0836	-1.7749
3.1	-3.4185	-2.8356	-2.5360
3.16	-3.6841	-3.1151	-2.8195
3.18	-3.7436	-3.1775	-2.8853
3.2	-3.7881	-3.2249	-2.9361
3.22	-3.8169	-3.2572	-2.9714
3.24	-3.8294	-3.2734	-2.9901
3.26	-3.8280	-3.2752	-2.9943
3.28	-3.8176	-3.2675	-2.9887
3.3	-3.8019	-3.2546	-2.9777
3.32	-3.7831	-3.2389	-2.9634
3.34	-3.7617	-3.2209	-2.9467

3.4	-3.6868	-3.1580	-2.8864
3.5	-3.4970	-2.9892	-2.7289
3.6	-3.2055	-2.7165	-2.4665
3.7	-2.9331	-2.4626	-2.2194
3.8	-2.6811	-2.2311	-1.9959
3.9	-2.4485	-2.0163	-1.7920
4.2	-1.8928	-1.5154	-1.3115
4.5	-1.4852	-1.1519	-0.9708
4.8	-1.1878	-0.8888	-0.7261
5.1	-0.9275	-0.6670	-0.5163
5.4	-0.7230	-0.4929	-0.3537
5.7	-0.5669	-0.3598	-0.2323
6	-0.4580	-0.2714	-0.1537

Table S2. Binding energy, ΔE, vs. (HO)H distance from the center of benzene ring, r, computed for (a) H_2O·bz, (b) H_2O·[bz·NCBr] and (c) H_2O·[bz·H_2O] systems in the T-shaped approach at the M06-2X/aug-cc-PVTZ level of theory.

r (Å)	ΔE (kcal/mol)		
	(a) H_2O·bz	(b) H_2O·[bz·NCBr]	(c) H_2O·[bz·H_2O]
1.8	1.0186	1.5986	1.4464
1.9	-0.9010	-0.3169	-0.4681
2	-2.1457	-1.5615	-1.7110
2.1	-2.9025	-2.3240	-2.4702
2.2	-3.2926	-2.7217	-2.8643
2.22	-3.3359	-2.7671	-2.9089
2.24	-3.3706	-2.8040	-2.9449
2.26	-3.3974	-2.8330	-2.9730
2.28	-3.4167	-2.8546	-2.9938
2.3	-3.4289	-2.8694	-3.0076
2.32	-3.4345	-2.8775	-3.0148
2.34	-3.4337	-2.8792	-3.0157
2.36	-3.4266	-2.8746	-3.0102
2.38	-3.4134	-2.8640	-2.9987
2.4	-3.3947	-2.8480	-2.9819
2.5	-3.2475	-2.7166	-2.8460
2.6	-3.0479	-2.5337	-2.6587
2.7	-2.8163	-2.3195	-2.4403
2.8	-2.5871	-2.1090	-2.2255
2.9	-2.3671	-1.9060	-2.0190
3	-2.1612	-1.7196	-1.8277
3.1	-1.9874	-1.5642	-1.6682
3.2	-1.8187	-1.4121	-1.5126
3.3	-1.6692	-1.2814	-1.3770
3.4	-1.5410	-1.1689	-1.2609

3.5	-1.4183	-1.0619	-1.1503
3.6	-1.3071	-0.9653	-1.0505
3.7	-1.1974	-0.8690	-0.9509
3.8	-1.1029	-0.7897	-0.8675
3.9	-1.0211	-0.7199	-0.7951
4.2	-0.7895	-0.5242	-0.5907
4.5	-0.6094	-0.3734	-0.4333
4.8	-0.4796	-0.2662	-0.3212
5.1	-0.3819	-0.1917	-0.2412
5.4	-0.3070	-0.1357	-0.1810
5.7	-0.2510	-0.0964	-0.1378
6	-0.2080	-0.0678	-0.1058

Table S3. Binding energy, ΔE, vs. Br distance from the center of benzene ring, r, computed for (a) NCBr·bz, (b) NCBr·[bz·H_2O] and (c) NCBr·[bz·NCBr] systems in the T-shaped approach at the M11/aug-cc-PVTZ level of theory.

r (Å)	ΔE (kcal/mol)		
	(a) NCBr·bz	(b) NCBr·[bz·H_2O]	(c) NCBr·[bz·NCBr]
1.8	178.1634	179.0501	179.4914
1.9	134.7102	135.5517	135.9873
2	100.1798	100.9890	101.4122
2.1	73.0510	73.8365	74.2441
2.2	52.0081	52.7750	53.1664
2.3	35.9179	36.6701	37.0451
2.4	23.8164	24.5556	24.9154
2.5	14.8875	15.6132	15.9604
2.6	8.4450	9.1548	9.4926
2.7	3.9216	4.6144	4.9443
2.8	0.8492	1.5245	1.8469
2.9	-1.1490	-0.4924	-0.1774
3	-2.3735	-1.7355	-1.4283
3.1	-3.0632	-2.4445	-2.1450
3.2	-3.3862	-2.7855	-2.4973
3.22	-3.4182	-2.8211	-2.5351
3.24	-3.4421	-2.8485	-2.5646
3.26	-3.4588	-2.8689	-2.5868
3.28	-3.4695	-2.8832	-2.6028
3.3	-3.4750	-2.8922	-2.6136
3.32	-3.4760	-2.8968	-2.6198
3.34	-3.4726	-2.8970	-2.6218
3.36	-3.4650	-2.8930	-2.6197
3.38	-3.4528	-2.8846	-2.6134
3.4	-3.4359	-2.8715	-2.6025
3.5	-3.2789	-2.7352	-2.4779

3.6	-3.0688	-2.5438	-2.2943
3.7	-2.8875	-2.3812	-2.1372
3.8	-2.6622	-2.1790	-1.9444
3.9	-2.3904	-1.9265	-1.7010
4.2	-1.8423	-1.4317	-1.2363
4.5	-1.4366	-1.0707	-0.8937
4.8	-1.0369	-0.7292	-0.5558
5.1	-0.8176	-0.5565	-0.4096
5.4	-0.6992	-0.4540	-0.3200
5.7	-0.5445	-0.3333	-0.2056
6	-0.4649	-0.2736	-0.1571

Table S4. Binding energy, ΔE, vs. (HO)H distance from the center of benzene ring, r, computed for (a) H_2O·bz, (b) H_2O·[bz·NCBr] and (c) H_2O·[bz·H_2O] systems in the T-shaped approach at the M11/aug-cc-PVTZ level of theory.

r (Å)	ΔE (kcal/mol)		
	(a) H_2O·bz	(b) H_2O·[bz·NCBr]	(c) H_2O·[bz·H_2O]
1.8	1.2958	1.8958	1.7560
1.9	-0.5876	0.0210	-0.1209
2	-1.7946	-1.1847	-1.3272
2.1	-2.5082	-1.9031	-2.0448
2.2	-2.8745	-2.2788	-2.4183
2.22	-2.9166	-2.3232	-2.4622
2.24	-2.9501	-2.3593	-2.4976
2.26	-2.9758	-2.3877	-2.5253
2.28	-2.9944	-2.4090	-2.5460
2.3	-3.0064	-2.4240	-2.5602
2.32	-3.0124	-2.4330	-2.5685
2.34	-3.0129	-2.4368	-2.5714
2.36	-3.0086	-2.4357	-2.5696
2.38	-2.9998	-2.4303	-2.5633
2.4	-2.9870	-2.4210	-2.5531
2.5	-2.8763	-2.3284	-2.4562
2.6	-2.7152	-2.1867	-2.3100
2.7	-2.5293	-2.0206	-2.1393
2.8	-2.3342	-1.8454	-1.9595
2.9	-2.1432	-1.6742	-1.7836
3	-1.9598	-1.5100	-1.6148
3.1	-1.7880	-1.3578	-1.4580
3.2	-1.6320	-1.2196	-1.3158
3.3	-1.4888	-1.0946	-1.1865
3.4	-1.3637	-0.9877	-1.0754
3.5	-1.2478	-0.8859	-0.9711
3.6	-1.1411	-0.7958	-0.8770

3.7	-1.0572	-0.7291	-0.8062
3.8	-0.9750	-0.6575	-0.7334
3.9	-0.8903	-0.5859	-0.6589
4.2	-0.7128	-0.4426	-0.5086
4.5	-0.5801	-0.3409	-0.3996
4.8	-0.4702	-0.2600	-0.3119
5.1	-0.3796	-0.1913	-0.2383
5.4	-0.3112	-0.1401	-0.1835
5.7	-0.2581	-0.1037	-0.1433
6	-0.2167	-0.0765	-0.1128

Table S5. Binding energy, ΔE, vs. Br distance from the center of benzene ring, r, computed for (a) NCBr·bz, (b) NCBr·[bz·H_2O] and (c) NCBr·[bz·NCBr] systems in the T-shaped approach at the *ω*B97X/aug-cc-PVTZ level of theory.

r (Å)	ΔE (kcal/mol)		
	(a) NCBr·bz	(b) NCBr·[bz·H_2O]	(c) NCBr·[bz·NCBr]
1.8	175.3732	176.1069	176.5309
1.9	133.0952	133.7828	134.2224
2.0	99.5822	100.2626	100.6763
2.1	73.4014	74.0654	74.4714
2.2	53.1105	53.7611	54.1529
2.3	37.5376	38.1807	38.5546
2.4	25.7282	26.3573	26.7185
2.5	16.8673	17.4921	17.8354
2.6	10.2907	10.9065	11.2322
2.7	5.5230	6.1250	6.4418
2.8	2.1188	2.7104	3.0154
2.9	-0.2519	0.3234	0.6182
3	-1.8109	-1.2474	-0.9638
3.1	-2.7924	-2.2453	-1.9682
3.2	-3.3884	-2.8577	-2.5900
3.3	-3.6221	-3.1052	-2.8494
3.32	-3.6395	-3.1265	-2.8713
3.34	-3.6518	-3.1423	-2.8872
3.36	-3.6614	-3.1551	-2.9007
3.38	-3.6675	-3.1643	-2.9114
3.4	-3.6701	-3.1696	-2.9188
3.42	-3.6681	-3.1720	-2.9221
3.44	-3.6603	-3.1689	-2.9196
3.46	-3.6477	-3.1587	-2.9125
3.48	-3.6266	-3.1414	-2.8981
3.5	-3.5993	-3.1174	-2.8758
3.6	-3.4077	-2.9421	-2.7098
3.7	-3.1807	-2.7312	-2.5059
3.8	-2.9344	-2.5030	-2.2855
3.9	-2.6796	-2.2642	-2.0561
4.2	-1.9774	-1.6113	-1.4233

4.5	-1.4363	-1.1118	-0.9434
4.8	-1.0542	-0.7699	-0.6156
5.1	-0.7901	-0.5406	-0.3993
5.4	-0.6198	-0.3978	-0.2682
5.7	-0.4925	-0.2955	-0.1751
6	-0.4110	-0.2338	-0.1226

Table S6. Binding energy, ΔE, vs. (HO)H distance from the center of benzene ring, r, computed for (a) H_2O·bz, (b) H_2O·[bz·NCBr] and (c) H_2O·[bz·H_2O] systems in the T-shaped approach at the *ω*B97X/aug-cc-PVTZ level of theory.

r (Å)	ΔE (kcal/mol)		
	(a) H_2O·bz	(b) H_2O·[bz·NCBr]	(c)H_2O·[bz·H_2O]
1.8	2.1563	2.6831	2.5516
1.9	0.0884	0.6210	0.4892
2	-1.3217	-0.7879	-0.9188
2.1	-2.2273	-1.6972	-1.8262
2.2	-2.7773	-2.2541	-2.3804
2.3	-3.0479	-2.5336	-2.6569
2.32	-3.0790	-2.5673	-2.6898
2.34	-3.1039	-2.5947	-2.7163
2.36	-3.1214	-2.6140	-2.7351
2.38	-3.1320	-2.6269	-2.7473
2.4	-3.1376	-2.6354	-2.7549
2.42	-3.1388	-2.6395	-2.7582
2.44	-3.1351	-2.6381	-2.7562
2.46	-3.1261	-2.6315	-2.7489
2.48	-3.1124	-2.6204	-2.7372
2.5	-3.0949	-2.6055	-2.7216
2.6	-2.9678	-2.4930	-2.6054
2.7	-2.7884	-2.3294	-2.4381
2.8	-2.5871	-2.1437	-2.2488
2.9	-2.3702	-1.9425	-2.0443
3	-2.1581	-1.7479	-1.8455
3.1	-1.9605	-1.5657	-1.6600
3.2	-1.7670	-1.3877	-1.4786
3.3	-1.5938	-1.2314	-1.3179
3.4	-1.4408	-1.0930	-1.1761
3.5	-1.2976	-0.9641	-1.0441
3.6	-1.1708	-0.8518	-0.9283
3.7	-1.0588	-0.7533	-0.8266
3.8	-0.9595	-0.6664	-0.7370
3.9	-0.8708	-0.5901	-0.6578
4.2	-0.6606	-0.4126	-0.4731
4.5	-0.5147	-0.2943	-0.3489
4.8	-0.4111	-0.2145	-0.2639

5.1	-0.3345	-0.1583	-0.2033
5.4	-0.2766	-0.1177	-0.1589
5.7	-0.2317	-0.0880	-0.1256
6	-0.1952	-0.0648	-0.0994

Table S7. Binding energy, ΔE, vs. Br distance from the center of benzene ring, r, computed for (a) NCBr·bz, (b) NCBr·[bz·H_2O] and (c) NCBr·[bz·NCBr] systems in the T-shaped approach at the *ω*B97XD/aug-cc-PVTZ level of theory.

r (Å)	ΔE (kcal/mol)	r (Å)	ΔE (kcal/mol)	
	(a) NCBr·bz		(b) NCBr·[bz·H_2O]	(c) NCBr·[bz·NCBr]
2.80	2.8148	2.86	1.8384	2.1076
2.90	0.4349	2.96	-0.1196	0.1463
3.00	-1.2346	3.06	-1.4250	-1.1731
3.10	-2.3213	3.16	-2.2844	-2.0394
3.20	-3.0199	3.26	-2.7824	-2.5454
3.30	-3.3925	3.36	-3.0331	-2.8025
3.32	-3.4386	3.38	-3.0628	-2.8329
3.34	-3.4759	3.4	-3.0881	-2.8596
3.36	-3.5066	3.42	-3.1119	-2.8837
3.38	-3.5327	3.44	-3.1301	-2.9048
3.40	-3.5551	3.46	-3.1398	-2.9179
3.42	-3.5752	3.48	-3.1412	-2.9194
3.44	-3.5912	3.5	-3.1354	-2.9154
3.46	-3.5973	3.52	-3.1267	-2.9085
3.48	-3.5926	3.54	-3.1122	-2.8963
3.50	-3.5843	3.56	-3.0985	-2.8835
3.60	-3.4932	3.66	-2.9844	-2.7757
3.70	-3.3441	3.76	-2.8310	-2.6298
3.80	-3.1623	3.86	-2.6511	-2.4579
3.90	-2.9626	3.96	-2.4642	-2.2764
4.00	-2.7599	4.06	-2.2766	-2.0955

Table S8. Binding energy, ΔE, vs. (HO)H distance from the center of benzene ring, r, computed for (a) H_2O·bz, (b) H_2O·[bz·NCBr] and (c) H_2O·[bz·H_2O] systems in the T-shaped approach at the *ω*B97XD/aug-cc-PVTZ level of theory.

r (Å)	ΔE (kcal/mol)	r (Å)	ΔE (kcal/mol)	
	(a) H_2O·bz		(b) H_2O·[bz·NCBr]	(c)H_2O·[bz·H_2O]
1.82	2.0859	1.86	1.6834	1.5698
1.92	0.0788	1.96	-0.0457	-0.1601
2.02	-1.2951	2.06	-1.2095	-1.3235
2.12	-2.1980	2.16	-1.9777	-2.0898
2.22	-2.7704	2.26	-2.4324	-2.5426
2.32	-3.0555	2.36	-2.6575	-2.7645
2.34	-3.0943	2.38	-2.6830	-2.7894
2.36	-3.1254	2.40	-2.7048	-2.8104
2.38	-3.1490	2.42	-2.7218	-2.8267

2.4	-3.1677	2.44	-2.7315	-2.8361
2.42	-3.1822	2.46	-2.7337	-2.8379
2.44	-3.1902	2.48	-2.7295	-2.8332
2.46	-3.1907	2.50	-2.7201	-2.8234
2.48	-3.1846	2.52	-2.7073	-2.8100
2.5	-3.1730	2.54	-2.6929	-2.7948
2.52	-3.1572	2.56	-2.6774	-2.7784
2.62	-3.0516	2.66	-2.5658	-2.6639
2.72	-2.8943	2.76	-2.4044	-2.4998
2.82	-2.7019	2.86	-2.2225	-2.3150
2.92	-2.5033	2.96	-2.0372	-2.1266
3.02	-2.3087	3.06	-1.8634	-1.9496

Table S9. Equilibrium distances r_{eq} (Å) and interaction energies ΔE_{XB} and ΔE_{HB} (kcal/mol) for NCBr·bz and H_2O·bz dimers in the 45° 'C approach' (Figure S13) and 'bond approach' (Figure S14).

	NCBr·bz	
	r_{eq}	ΔE_{XB}
'C approach'	4.12	-2.00
'bond approach'	4.02	-2.34
	**H(1)OH(2)·bz**[a]	
	r_{eq}	ΔE_{HB}
'C approach', H(1) up[b]	3.12	-2.29
'C approach', H(1) down[c]	3.24	-1.50
'bond approach', H(1) up[b]	3.06	-2.51
'bond approach', H(1) down[c]	3.16	-1.77

[a] H(1) and H(2) indicate the non-interacting and the interacting H atoms, respectively; [b]H(1) up indicates the H(1) points far from the benzene ring; [b]H(1) down indicates the H(1) points towards the benzene ring.

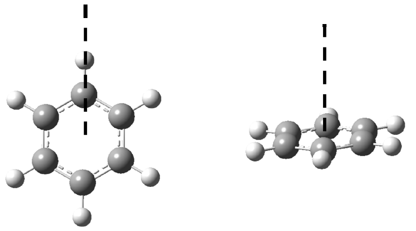

Figure S8. Top (left) and side (right) views of the 45° approach of NCBr or H_2O towards the center of benzene ring along the line whose projection on the benzene plane crosses one carbon atom ('C approach').

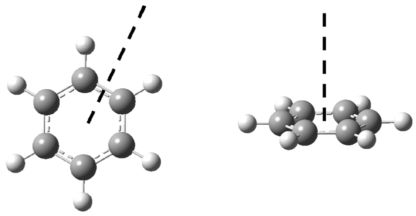

Figure S9. Top (left) and side (right) views of the 45° approach of NCBr or H_2O towards the center of benzene ring along the line whose projection on the benzene plane crosses the center of a CC bond ('bond approach').

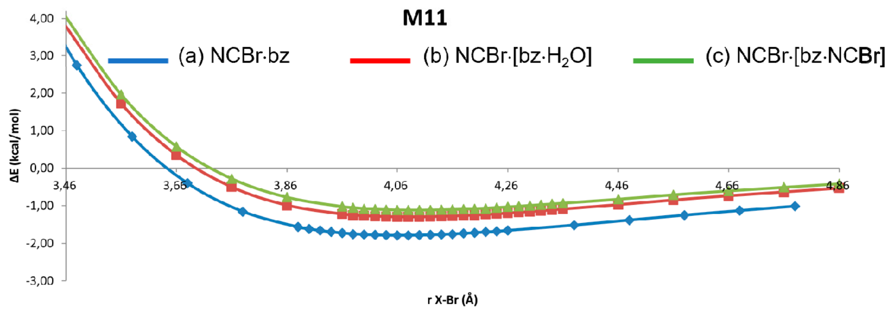

Figure S10. Binding energy curves, ΔE, vs. Br distance from the center of benzene ring, r, computed for (a) NCBr·bz (blue line), (b) NCBr·[bz·H_2O] (red line) and (c) NCBr·[bz·NCBr] (green line) systems in the perpendicular approach at the M11/aug-cc-PVTZ level of theory.

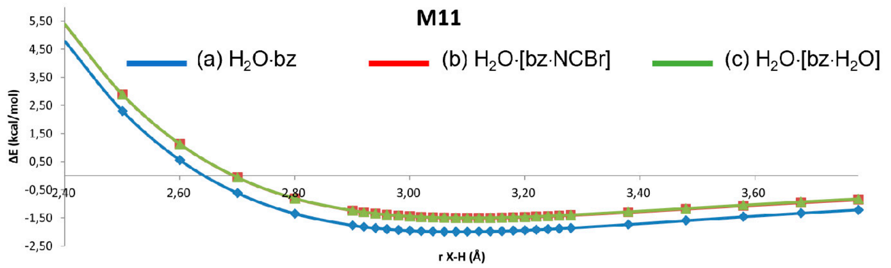

Figure S11. Binding energy curves, ΔE, vs. (HO)H distance from the center of benzene ring, r, computed for (a) H_2O·bz (blue line), (b) H_2O·[bz·NCBr] (red line) and (c) H_2O·[bz·H_2O] (green line) systems in the perpendicular approach at the M11/aug-cc-PVTZ level of theory.

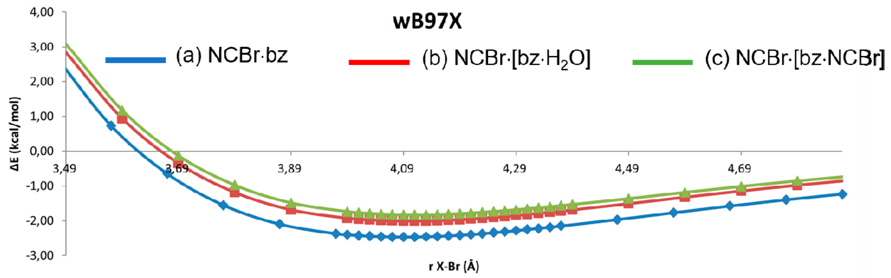

Figure S12. Binding energy curves, ΔE, vs. Br distance from the center of benzene ring, r, computed for (a) NCBr·bz (blue line), (b) NCBr·[bz·H_2O] (red line) and (c) NCBr·[bz·NCBr] (green line) systems in the perpendicular approach at the *ω*B97X/aug-cc-PVTZ level of theory.

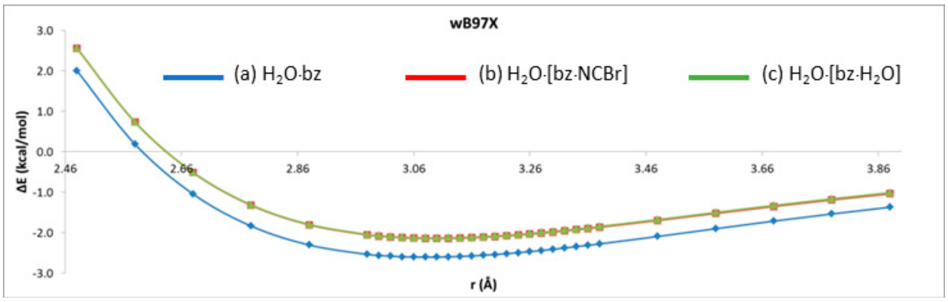

Figure S13. Binding energy curves, ΔE, vs. (HO)H distance from the center of benzene ring, r, computed for (a) H_2O·bz (blue line), (b) H_2O·[bz·NCBr] (red line) and (c) H_2O·[bz·H_2O] (green line) systems in the perpendicular approach at the *ω*B97X/aug-cc-PVTZ level of theory.

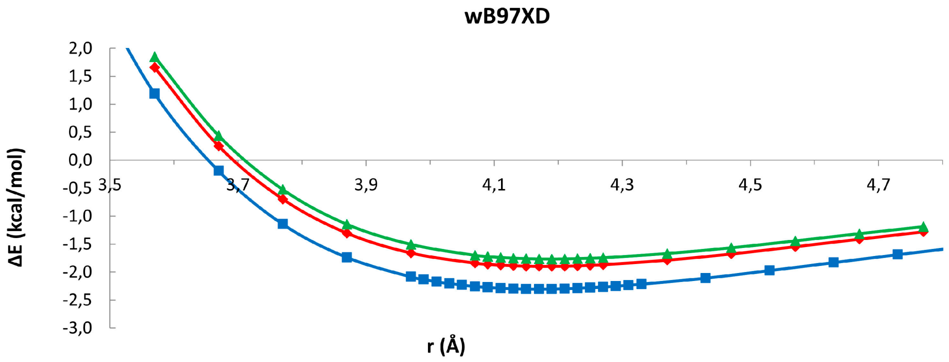

Figure S14. Binding energy curves, ΔE, vs. Br distance from the center of benzene ring, r, computed for (a) NCBr·bz (blue line), (b) NCBr·[bz·H_2O] (red line) and (c) NCBr·[bz·NCBr] (green line) systems in the perpendicular approach at the ωB97XD/aug-cc-PVTZ level of theory.

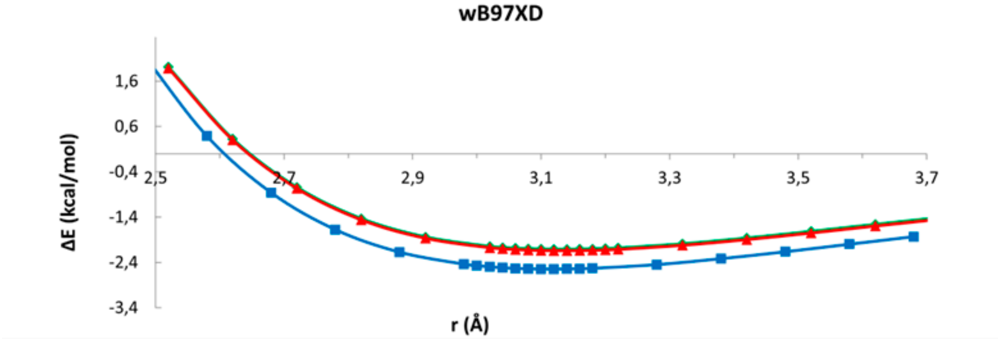

Figure S15. Binding energy curves, ΔE, vs. (HO)H distance from the center of benzene ring, r, computed for (a) H_2O·bz (blue line), (b) H_2O·[bz·NCBr] (red line) and (c) H_2O·[bz·H_2O] (green line) systems in the perpendicular approach at the ωB97XD/aug-cc-PVTZ level of theory.

Table S10. Binding energy, ΔE, vs. Br distance from the center of benzene ring, r, computed for (a) NCBr·bz, (b) NCBr·[bz·H_2O] and (c) NCBr·[bz·NCBr] systems in the perpendicular approach at the M06-2X/aug-cc-PVTZ level of theory.

r (Å)	ΔE (kcal/mol)	r (Å)	ΔE (kcal/mol)	
	(a) NCBr·bz		(b) NCBr·[bz·H_2O]	(c) NCBr·[bz·NCBr]
3.20	12.1989	3.34	6.5090	6.7773
3.30	7.4943	3.44	3.4488	3.6984
3.40	4.0218	3.54	1.2968	1.5331
3.50	1.5405	3.64	-0.1514	0.0737
3.60	-0.1571	3.74	-1.0591	-0.8454
3.70	-1.2542	3.84	-1.5863	-1.3840
3.80	-1.9043	3.86	-1.6545	-1.4553
3.82	-1.9952	3.88	-1.7123	-1.5160
3.84	-2.0744	3.90	-1.7602	-1.5668
3.86	-2.1419	3.92	-1.7984	-1.6077
3.88	-2.1981	3.94	-1.8276	-1.6386
3.90	-2.2438	3.96	-1.8492	-1.6613
3.92	-2.2797	3.98	-1.8644	-1.6777
3.94	-2.3065	4.00	-1.8739	-1.6889
3.96	-2.3251	4.02	-1.8777	-1.6951

3.98	-2.3370	4.04	-1.8765	-1.6968
4.00	-2.3433	4.06	-1.8712	-1.6946
4.02	-2.3448	4.08	-1.8626	-1.6884
4.04	-2.3420	4.10	-1.8506	-1.6778
4.06	-2.3353	4.12	-1.8352	-1.6631
4.08	-2.3252	4.14	-1.8164	-1.6449
4.10	-2.3117	4.16	-1.7943	-1.6236
4.12	-2.2947	4.18	-1.7692	-1.5993
4.14	-2.2740	4.20	-1.7409	-1.5726
4.16	-2.2498	4.22	-1.7100	-1.5439
4.18	-2.2222	4.32	-1.5371	-1.3814
4.20	-2.1913	4.42	-1.3647	-1.2169
4.30	-2.0085	4.52	-1.1965	-1.0611
4.40	-1.8268	4.62	-1.0409	-0.9078
4.50	-1.6446	4.72	-0.9088	-0.7878
4.60	-1.4752			
4.70	-1.3273			
4.80	-1.2007			
4.90	-1.0920			
5.00	-0.9980			
5.10	-0.9099			

Table S11. Binding energy, ΔE, vs. (HO)H distance from the center of benzene ring, r, computed for (a) H_2O·bz, (b) H_2O·[bz·NCBr] and (c) H_2O·[bz·H_2O] systems in the perpendicular approach at the M06-2X/aug-cc-PVTZ level of theory.

r (Å)	ΔE (kcal/mol)	r (Å)	ΔE (kcal/mol)	
	(a) H_2O·bz		(b) H_2O·[bz·NCBr]	(c) H_2O·[bz·H_2O]
1.80	48.6053	2.38	5.0622	5.0788
2.00	25.4302	2.48	2.5218	2.5362
2.20	11.6484	2.58	0.7180	0.7313
2.40	3.9324	2.68	-0.5104	-0.4973
2.50	1.5608	2.78	-1.2977	-1.2839
2.60	-0.1063	2.88	-1.7553	-1.8773
2.70	-1.2241	2.90	-1.8159	-1.9356
2.80	-1.9237	2.92	-1.8680	-1.9854
2.90	-2.3120	2.94	-1.9121	-2.0272
2.92	-2.3610	2.96	-1.9487	-2.0614
2.94	-2.4020	2.98	-1.9783	-2.0886
2.96	-2.4355	3.00	-2.0012	-2.1092
2.98	-2.4619	3.02	-2.0181	-2.1237
3.00	-2.4817	3.04	-2.0295	-2.1327
3.02	-2.4954	3.06	-2.0359	-2.1367
3.04	-2.5035	3.08	-2.0378	-2.1363
3.06	-2.5066	3.10	-2.0356	-2.1317
3.08	-2.5052	3.12	-2.0296	-2.1234

3.10	-2.4996	3.14	-2.0201	-2.1116
3.12	-2.4902	3.16	-2.0074	-2.0966
3.14	-2.4773	3.18	-1.9917	-2.0787
3.16	-2.4612	3.20	-1.9733	-2.0581
3.18	-2.4421	3.22	-1.9526	-2.0351
3.20	-2.4203	3.24	-1.9298	-2.0102
3.22	-2.3961	3.26	-1.9054	-1.9836
3.24	-2.3699	3.36	-1.7640	-1.7410
3.26	-2.3420	3.46	-1.6052	-1.5797
3.28	-2.3125	3.56	-1.4459	-1.4186
3.30	-2.2816	3.66	-1.2947	-1.2653
3.40	-2.1139	3.76	-1.1591	-1.1276
3.50	-1.9363			
3.60	-1.7631			
3.70	-1.6010			
3.80	-1.4558			
3.90	-1.3233			
4.00	-1.2082			
4.20	-1.0086			
4.40	-0.8454			
4.60	-0.7037			
4.80	-0.5887			
5.00	-0.4868			

Table S12. Binding energy, ΔE, vs. Br distance from the center of benzene ring, r, computed for (a) NCBr·bz, (b) NCBr·[bz·H_2O] and (c) NCBr·[bz·NCBr] systems in the perpendicular approach at the M11/aug-cc-PVTZ level of theory.

r (Å)	ΔE (kcal/mol)	r (Å)	ΔE (kcal/mol)	
	(a) NCBr·bz		(b) NCBr·[bz·H_2O]	(c) NCBr·[bz·NCBr]
3.38	5.5218	3.46	3.7942	4.0425
3.48	2.7394	3.56	1.7195	1.9557
3.58	0.8373	3.66	0.3498	0.5755
3.68	-0.4006	3.76	-0.5024	-0.2790
3.78	-1.1540	3.86	-0.9880	-0.7736
3.88	-1.5670	3.96	-1.2232	-1.0224
3.90	-1.6188	3.98	-1.2486	-1.0510
3.92	-1.6623	4.00	-1.2682	-1.0741
3.94	-1.6982	4.02	-1.2826	-1.0920
3.96	-1.7271	4.04	-1.2924	-1.1049
3.98	-1.7497	4.06	-1.2980	-1.1131
4.00	-1.7665	4.08	-1.2997	-1.1169
4.02	-1.7782	4.10	-1.2981	-1.1169
4.04	-1.7853	4.12	-1.2934	-1.1139
4.06	-1.7882	4.14	-1.2860	-1.1084

4.08	-1.7873	4.16	-1.2761	-1.1007
4.10	-1.7829	4.18	-1.2640	-1.0910
4.12	-1.7754	4.20	-1.2499	-1.0792
4.14	-1.7651	4.22	-1.2340	-1.0654
4.16	-1.7522	4.24	-1.2165	-1.0496
4.18	-1.7370	4.26	-1.1976	-1.0321
4.20	-1.7197	4.28	-1.1775	-1.0133
4.22	-1.7007	4.30	-1.1565	-0.9936
4.24	-1.6803	4.32	-1.1348	-0.9733
4.26	-1.6587	4.34	-1.1126	-0.9527
4.38	-1.5154	4.36	-1.0901	-0.9319
4.48	-1.3829	4.46	-0.9739	-0.8267
4.58	-1.2505	4.56	-0.8500	-0.7094
4.68	-1.1297	4.66	-0.7368	-0.6060
4.78	-1.0117	4.76	-0.6358	-0.5108
		4.86	-0.5343	-0.4172

Table S13. Binding energy, ΔE, vs. (HO)H distance from the center of benzene ring, r, computed for (a) H_2O·bz, (b) H_2O·[bz·NCBr] and (c) H_2O·[bz·H_2O] systems in the perpendicular approach at the M11/aug-cc-PVTZ level of theory.

r (Å)	ΔE (kcal/mol)		
	(a) H_2O·bz	(b) H_2O·[bz·NCBr]	(c) H_2O·[bz·H_2O]
2.40	4.7774	5.3742	5.3754
2.50	2.2983	2.8843	2.8841
2.60	0.5552	1.1278	1.1277
2.70	-0.6147	-0.0580	-0.0565
2.80	-1.3489	-0.8093	-0.8057
2.90	-1.7630	-1.2408	-1.2356
2.92	-1.8161	-1.2974	-1.2919
2.94	-1.8610	-1.3458	-1.3400
2.96	-1.8981	-1.3865	-1.3805
2.98	-1.9283	-1.4203	-1.4140
3.00	-1.9519	-1.4476	-1.4409
3.02	-1.9697	-1.4690	-1.4620
3.04	-1.9820	-1.4850	-1.4776
3.06	-1.9893	-1.4961	-1.4882
3.08	-1.9921	-1.5028	-1.4944
3.10	-1.9909	-1.5055	-1.4965
3.12	-1.9861	-1.5045	-1.4949
3.14	-1.9779	-1.5004	-1.4900
3.16	-1.9669	-1.4933	-1.4822
3.18	-1.9532	-1.4836	-1.4718
3.20	-1.9373	-1.4717	-1.4592
3.22	-1.9193	-1.4577	-1.4445

3.24	-1.8996	-1.4420	-1.4282
3.26	-1.8784	-1.4248	-1.4103
3.28	-1.8559	-1.4063	-1.3910
3.38	-1.7295	-1.2997	-1.2810
3.48	-1.5928	-1.1818	-1.1601
3.58	-1.4579	-1.0652	-1.0409
3.68	-1.3300	-0.9548	-0.9274
3.78	-1.2099	-0.8519	-0.8221

Table S14. Binding energy, ΔE, vs. Br distance from the center of benzene ring, r, computed for (a) NCBr·bz, (b) NCBr·[bz·H_2O] and (c) NCBr·[bz·NCBr] systems in the perpendicular approach at the ωB97X/aug-cc-PVTZ level of theory.

r (Å)	ΔE (kcal/mol)	r (Å)	ΔE (kcal/mol)	
	(a) NCBr·bz		(b) NCBr·[bz·H_2O]	(c) NCBr·[bz·NCBr]
3.47	2.7645	3.49	2.8320	3.0767
3.57	0.7338	3.59	0.9359	1.1677
3.67	-0.6474	3.69	-0.3520	-0.1344
3.77	-1.5518	3.79	-1.1845	-0.9762
3.87	-2.0975	3.89	-1.6838	-1.4875
3.97	-2.3714	3.99	-1.9271	-1.7389
3.99	-2.4019	4.01	-1.9535	-1.7678
4.01	-2.4256	4.03	-1.9730	-1.7897
4.03	-2.4428	4.05	-1.9876	-1.8070
4.05	-2.4551	4.07	-1.9977	-1.8194
4.07	-2.4630	4.09	-2.0028	-1.8271
4.09	-2.4667	4.11	-2.0036	-1.8296
4.11	-2.4658	4.13	-2.0000	-1.8271
4.13	-2.4599	4.15	-1.9924	-1.8205
4.15	-2.4498	4.17	-1.9809	-1.8099
4.17	-2.4353	4.19	-1.9651	-1.7955
4.19	-2.4172	4.21	-1.9462	-1.7776
4.21	-2.3958	4.23	-1.9240	-1.7567
4.23	-2.3705	4.25	-1.8990	-1.7346
4.25	-2.3430	4.27	-1.8722	-1.7101
4.27	-2.3138	4.29	-1.8431	-1.6837
4.29	-2.2831	4.31	-1.8134	-1.6555
4.31	-2.2513	4.33	-1.7830	-1.6262
4.33	-2.2183	4.35	-1.7515	-1.5959
4.35	-2.1843	4.37	-1.7195	-1.5649
4.37	-2.1498	4.39	-1.6863	-1.5332
-1.97	-1.9655	4.49	-1.5089	-1.3654
-1.77	-1.7686	4.59	-1.3229	-1.1852
-1.58	-1.5766	4.69	-1.1445	-1.0147
-1.40	-1.3966	4.79	-0.9782	-0.8552

-1.23	-1.2314	4.89	-0.8264	-0.7096

Table S15. Binding energy, ΔE, vs. (HO)H distance from the center of benzene ring, r, computed for (a) H_2O·bz, (b) H_2O·[bz·NCBr] and (c) H_2O·[bz·H_2O] systems in the perpendicular approach at the ωB97X/aug-cc-PVTZ level of theory.

r (Å)	ΔE (kcal/mol)		
	(a) H_2O·bz	(b) H_2O·[bz·NCBr]	(c) H_2O·[bz·H_2O]
2.48	2.0032	2.5586	2.5679
2.58	0.1876	0.7316	0.7403
2.68	-1.0439	-0.5138	-0.5048
2.78	-1.8354	-1.3208	-1.3107
2.88	-2.3032	-1.8049	-1.7938
2.98	-2.5355	-2.0540	-2.0418
3.00	-2.5605	-2.0824	-2.0700
3.02	-2.5795	-2.1049	-2.0922
3.04	-2.5929	-2.1217	-2.1087
3.06	-2.6008	-2.1330	-2.1196
3.08	-2.6034	-2.1390	-2.1252
3.10	-2.6012	-2.1403	-2.1262
3.12	-2.5950	-2.1377	-2.1233
3.14	-2.5855	-2.1318	-2.1169
3.16	-2.5727	-2.1225	-2.1073
3.18	-2.5567	-2.1099	-2.0945
3.20	-2.5379	-2.0946	-2.0788
3.22	-2.5167	-2.0768	-2.0606
3.24	-2.4930	-2.0566	-2.0400
3.26	-2.4670	-2.0339	-2.0170
3.28	-2.4388	-2.0092	-1.9918
3.30	-2.4087	-1.9825	-1.9648
3.32	-2.3770	-1.9544	-1.9363
3.34	-2.3442	-1.9251	-1.9066
3.36	-2.3105	-1.8947	-1.8759
3.38	-2.2757	-1.8632	-1.8441
3.48	-2.0921	-1.6964	-1.6754
3.58	-1.9007	-1.5212	-1.4987
3.68	-1.7120	-1.3489	-1.3244
3.78	-1.5352	-1.1877	-1.1615
3.88	-1.3694	-1.0370	-1.0089

Table S16. Binding energy, ΔE, vs. Br distance from the center of benzene ring, r, computed for (a) NCBr·bz, (b) NCBr·[bz·H_2O] and (c) NCBr·[bz·NCBr] systems in the perpendicular approach at the ωB97XD/aug-cc-PVTZ level of theory.

r (Å)	ΔE (kcal/mol)	r (Å)	ΔE (kcal/mol)	
	(a) NCBr·bz		(b) NCBr·[bz·H_2O]	(c) NCBr·[bz·NCBr]
3.47	3.1997	3.57	1.6572	1.8516
3.57	1.1912	3.67	0.2548	0.4382
3.67	-0.1922	3.77	-0.6947	-0.5241
3.77	-1.1422	3.87	-1.3026	-1.1454

3.87	-1.7358	3.97	-1.6579	-1.5044
3.97	-2.0838	4.07	-1.8401	-1.7005
3.99	-2.1310	4.09	-1.8602	-1.7222
4.01	-2.1707	4.11	-1.8755	-1.7396
4.03	-2.2032	4.13	-1.8865	-1.7518
4.05	-2.2306	4.15	-1.8941	-1.7606
4.07	-2.2540	4.17	-1.8983	-1.7664
4.09	-2.2734	4.19	-1.8981	-1.7676
4.11	-2.2881	4.21	-1.8952	-1.7651
4.13	-2.2975	4.23	-1.8889	-1.7596
4.15	-2.3029	4.25	-1.8797	-1.7521
4.17	-2.3040	4.27	-1.8681	-1.7441
4.19	-2.3022	4.37	-1.7872	-1.6682
4.21	-2.2974	4.47	-1.6763	-1.5658
4.23	-2.2886	4.57	-1.5453	-1.4428
4.25	-2.2776	4.67	-1.4117	-1.3136
4.27	-2.2648	4.77	-1.2799	-1.1885
4.29	-2.2498			
4.31	-2.2328			
4.33	-2.2138			
4.43	-2.1069			
4.53	-1.9714			
4.63	-1.8292			
4.73	-1.6868			
4.83	-1.5498			

Table S17. Binding energy, ΔE, vs. (HO)H distance from the center of benzene ring, r, computed for (a) H_2O·bz, (b) H_2O·[bz·NCBr] and (c) H_2O·[bz·H_2O] systems in the perpendicular approach at the ωB97XD/aug-cc-PVTZ level of theory.

r (Å)	ΔE (kcal/mol)	r (Å)	ΔE (kcal/mol)	
	(a) H_2O·bz		(b) H_2O·[bz·NCBr]	(c) H_2O·[bz·H_2O]
2.48	2.2128	2.52	1.8852	1.9131
2.58	0.3886	2.62	0.2994	0.3266
2.68	-0.8613	2.72	-0.7700	-0.7429
2.78	-1.6780	2.82	-1.4611	-1.4332
2.88	-2.1750	2.92	-1.8678	-1.8392
2.98	-2.4403	3.02	-2.0768	-2.0474
3.00	-2.4724	3.04	-2.1002	-2.0705
3.02	-2.4984	3.06	-2.1177	-2.0878
3.04	-2.5188	3.08	-2.1298	-2.0996
3.06	-2.5337	3.10	-2.1373	-2.1068
3.08	-2.5431	3.12	-2.1411	-2.1102
3.10	-2.5475	3.14	-2.1418	-2.1107
3.12	-2.5481	3.16	-2.1395	-2.1081
3.14	-2.5457	3.18	-2.1339	-2.1023

3.16	-2.5404	3.20	-2.1256	-2.0937
3.18	-2.5319	3.22	-2.1147	-2.0827
3.28	-2.4497	3.32	-2.0232	-1.9897
3.38	-2.3191	3.42	-1.8975	-1.8628
3.48	-2.1660	3.52	-1.7518	-1.7155
3.58	-1.9991	3.62	-1.5977	-1.5602
3.68	-1.8323	3.72	-1.4477	-1.4088
